# 5-Aminolevulinic Acid Thins Pear Fruits by Inhibiting Pollen Tube Growth via Ca^2+^-ATPase-Mediated Ca^2+^ Efflux

**DOI:** 10.3389/fpls.2016.00121

**Published:** 2016-02-09

**Authors:** Yuyan An, Jie Li, Chunhui Duan, Longbo Liu, Yongping Sun, Rongxiang Cao, Liangju Wang

**Affiliations:** ^1^College of Horticulture, Nanjing Agricultural UniversityNanjing, China; ^2^Nanjing Institute of Agricultural SciencesNanjing, China

**Keywords:** 5-aminolevulinic acid (ALA), Ca^2+^-ATPase, calcium, fruit set, pollen germination

## Abstract

Chemical fruit thinning has become a popular practice in modern fruit orchards for achieving high quality fruits, reducing costs of hand thinning and promoting return bloom. However, most of the suggested chemical thinners are often concerned for their detrimental effects and environmental problems. 5-Aminolevulic acid (ALA) is a natural, nontoxic, biodegradable, and environment-friendly plant growth regulator. One of its outstanding roles is improving plant photosynthesis and fruit quality. Here, results showed that applying 100–200 mg/L ALA at full bloom stage significantly reduced pear fruit set. Both *in vivo* and *in vitro* studies showed that ALA significantly inhibited pollen germination and tube growth. ALA decreased not only cytosolic Ca^2+^ concentration ([Ca^2+^]_cyt_) but also “tip-focused” [Ca^2+^]_cyt_ gradient, indicating that ALA inhibited pollen tube growth by down-regulating calcium signaling. ALA drastically enhanced pollen Ca^2+^-ATPase activity, suggesting that ALA-induced decrease of calcium signaling probably resulted from activating calcium pump. The significant negative correlations between Ca^2+^-ATPase activity and pollen germination or pollen tube length further demonstrated the critical role of calcium pump in ALA's negative effect on pollen germination. Taken together, our results suggest that ALA at low concentrations is a potential biochemical thinner, and it inhibits pollen germination and tube growth via Ca^2+^ efflux by activating Ca^2+^-ATPase, thereby thinning fruits by preventing fertilization.

## Introduction

Many species of fruit trees, including pear, often bear an abundance of flowers, and produce a surplus of fruits that the tree is unable to support, resulting in many low quality fruits (Bangerth, 2000). Therefore, flower or fruit thinning is necessary for increasing fruit size and maintaining regular bearing (Costa et al., 1995). Hand thinning is a common practice in many orchards. However, this practice involves intensive labor and increases the production costs, especially in a large scale commercial orchard (Bangerth, 2000). The increasing costs of hand thinning makes chemical thinning as an alternative. Nowadays, chemical thinning, especially with bioregulators (plant growth regulators as well as endogenous plant hormones), has become a popular practice in many orchards for achieving high quality fruits, reducing costs of hand thinning, and promoting return bloom (Gonkiewicz et al., 2011). Several chemical agents, such as NAA and its amide (Stopar, 2002), 6-BA (Bound, 2006), ethephon (Jones et al., 1989), carbaryl (Bound, 2006), and ammonium thiosulfate (Janoudi and Flore, 2005), have been reported to be used in fruit thinning. However, many chemicals presently used as thinners are either unsatisfactory or unreliable or both with regard to their thinning efficiencies. For example, carbaryl was a standard thinner for apple production in many countries, but it caused severe environmental problems and hence was banned in some countries (Bound, 2006). Ethephon is a chemical thinner of Japanese pear, but it was reported to cause fruit weight decrease by 10–20% (Burge et al., 1991). Therefore, new effective flower or fruit thinning techniques, which have no detrimental effects and meet modern environmental and food quality guidelines, are required.

5-aminolevulinic acid (ALA) is an essential precursor in tetrapyrrole biosynthesis in all organisms. Increasing evidence indicates that ALA is not only an important intermediate in biological metabolism but also a vital plant growth regulator which regulates several key physiological processes such as promoting plant growth/yields and increasing plant stress tolerance at low concentrations (Akram and Ashraf, 2013). In addition, it has been well-documented that exogenous ALA over a very wide range of concentrations improved fruit weight and quality of many fruit trees (Iwai et al., 2005; Al-Khateeb et al., 2006; Al-Qurashi and Awad, 2011; Shen et al., 2011; Xie et al., 2013a,b; Feng et al., 2015). Shen et al. (2011) reported that ALA at high concentrations (600–1200 mg/L) significantly reduced pear fruit set and improved fruit quality, indicating ALA at high concentration is effective in thinning pear fruits. Since it is a natural substance in all organisms, ALA is expected to be a promising choice for chemical fruit thinning. However, ALA is expensive in current international market, and the high cost blocks its use in fruit thinning at such high concentrations. Moreover, thinning effect of ALA at high concentrations resulted from its damage effect on floral organ including stigma (Shen et al., 2011), similar to its role as a natural photodynamic herbicide/insecticide (Sasikala et al., 1994). Therefore, whether ALA at low concentrations can thin fruits and how it works are the important questions that need to be solved before its utilization as a reliable and competitive biochemical thinner.

Pollen germination and tube growth are key events in plant sexual reproduction. Inhibition of pollen germination and tube growth has been reported as a thinning mechanism for some chemical thinners (Costa et al., 1995; Hiratsuka et al., 2003). Pollen tube growth, which is central to the vital process of sexual reproduction, is tightly regulated (Franklin-Tong, 1999). It has been well established that Ca^2+^ plays a key role in regulating pollen tube growth (Lazzaro et al., 2005; Wu et al., 2010). Inhibition of Ca^2+^ uptake results in rapid arrest of pollen tube growth (Iwano et al., 2009). Moreover, not only the cytosolic free Ca^2+^ concentration ([Ca^2+^]_cyt_), but also the tip-specific [Ca^2+^]_cyt_ gradient play pivotal roles in controlling pollen tube elongation (Guan et al., 2013). The influx of extracellular Ca^2+^ and the efflux of intracellular Ca^2+^ are involved in the regulation of the Ca^2+^ gradient (Franklin-Tong, 1999). Ca^2+^ influx is mainly achieved through active Ca^2+^ channels at the pollen tube, while efflux occurs through Ca^2+^ pumps (Ca^2+^-ATPase) and antiporters (Franklin-Tong, 1999; Schiøtt et al., 2004). Since ALA at low concentrations decreased [Ca^2+^]_cyt_ in guard cells (Chen et al., 2014), we hypothesized that ALA at low concentrations inhibits pollen tube growth through Ca^2+^-ATPase-mediated Ca^2+^ efflux.

To test our hypothesis, we sprayed low concentrations of ALA (50–200 mg/L) to pear flowers at different stages of bloom. We found that applying 100 mg/L ALA at 50–75% bloom significantly reduced pear fruit set. Both *in vivo* and *in vitro* studies showed that ALA significantly inhibited pollen germination and pollen tube length. To explore the mechanisms of ALA, we observed [Ca^2+^]_cyt_ in pollens using confocal laser scanning microscopy, and found that ALA decreased both [Ca^2+^]_cyt_ and “tip-focused” [Ca^2+^]_cyt_ gradient. Ca^2+^-ATPase may contribute to ALA-induced reduction of [Ca^2+^]_cyt_ in pollen tubes. Our results suggest that ALA at low concentrations can be used for pear fruit thinning, providing a safe and efficient fruit thinner for modern fruit production.

## Materials and methods

### Field experiments and survey of fruit set

Field experiments were conducted in the Horticultural Experimental Station, Nanjing Agricultural University, Jiangsu Province, China. A dozen cultivars of pear (*Pyrus pyrifolia* Nakai), including “Cuiguan,” “Akemizu,” “Suisho” et al., were mixed planted in the brown yellow soil at a spacing of 4 × 5 m. Flowers of each cultivar were naturally cross-pollinated. Twelve-years-old “Cuiguan” trees were used for the experiments. In March 2012, five “Cuiguan” trees uniform in height, stem circumference, and blossom density were chosen and four representative branches per tree were tagged in the lower half of the tree canopy before anthesis. In each tree, exogenous ALA at 0, 50, 100, and 200 mg/L were sprayed evenly to the tagged branches, respectively, at 75% bloom (75% of the blossoms open). Fruit set for each treatment was recorded 6 weeks later to evaluate the appropriate concentration of exogenous ALA in fruit thinning. As both 100 and 200 mg/L ALA could significantly reduce the later fruit set, 100 mg/L ALA was chosen for the following experiments. To determine the optimum time for ALA application, in March 2013, another 15 “Cuiguan” trees uniform in height, stem circumference, and blossom density were chosen and two representative branches per tree were tagged in the lower half of the tree canopy before anthesis. The selected trees were randomly divided into three groups, five trees in each group. For the first group, 0 and 100 mg/L ALA were sprayed evenly to the tagged branches, respectively, at 25% bloom (25% of the blossoms open). The same treatments were set in the second group at 50% bloom (50% of the blossoms open) and in the third group at 75% bloom, respectively. Fruit set for each treatment was recorded once a week until final fruit number was determined. Fruit set percentage was calculated as the ratio of the fruit number to the flower number.

### *In vivo* pollen tube growth

In early March 2013, “Cuiguan” flower twigs uniform in length, stem circumference, and blossom density were detached, transferred to the laboratory and cultured with 10% sucrose solution, in a growth chamber at 25°C under a photosynthetic photon flux density (PPFD) of 50 μmol·m^−2^·s^−1^ in 12 h light/12 h dark cycles. In late March, flower twigs were randomly separated into two identical groups for two independent experiments, in each of which, the twigs were further divided into eight subgroups for different treatments. In experiment I, flowers of four subgroups were sprayed with 100 mg/L ALA solution, and then hand pollinated 2, 8, 12, and 24 h later, respectively. Flowers of the left four subgroups, which were sprayed with distilled water and then hand pollinated 2, 8, 12, and 24 h later, were set as controls. In experiment II, flowers of four subgroups were first hand pollinated and then sprayed 100 mg/L ALA 2, 8, 12, and 24 h later, respectively. Flowers of the left four subgroups, which were hand pollinated and then sprayed with distilled water 2, 8, 12, and 24 h later, were set as controls. In these two experiments, “Cuiguan” flowers were hand pollinated with “Akemizu” pollens.

Pistils at 48 h post-pollination were collected and fixed in FAA (formalin: acetic acid: ethanol 70%, 1:1:18, v/v). After rinsing in water two to three times, the pistils were macerated in 2 M NaOH at 60°C for 2 h, and then stained with 0.1% (w/v) aniline blue (AB) (in 0.15 M K_2_HPO_4_, pH 11) for 2 h. The pistils were mounted on slides and observed under a stereomicroscope (Olympus MVX10, Japan). Subsequently, the distance between the center of the stigma and the tip of the pollen tube (pollen tube length) was measured. At least 15 styles from a population of 3 different plants were used for each treatment and experiments were repeated three times.

### *In vitro* pollen germination and tube growth

Fresh mature pollen from “Cuiguan” pear blossoms, was collected from the Horticultural Experimental Station and preserved by drying in air at ambient temperature for 12 h and then stored in silica gel at −20°C. Pollen was germinated in simplified culture, containing 10% sucrose and 0.01% H_3_BO_3_, and incubated at 25°C at 100% humidity, in the dark. ALA at concentrations of 0, 5, 10, 15, and 20 mg/L were added separately to the basal medium to examine the effect of ALA on pollen germination and tube growth. CaCl_2_ at 10^−2^, 10^−3^, 10^−4^, 10^−5^ M, and EGTA (ethylene glycol tetraacetic acid, a Ca-chelating agent) at 10^−5^, 10^−4^, and 10^−3^ M were added separately to the basal medium to determine the suitable concentration of CaCl_2_ and EGTA for the following experiments. Then, ALA and CaCl_2_ at appropriate concentration were applied together to the basal medium with or without suitable EGTA to determine the interaction between ALA, Ca^2+^, and EGTA. Pollen germination and pollen tube length were observed 3 h after treatment under a light microscope (Nikon TE100, 400 ×), and measured using a fitted camera (MShot Digital Imaging System) and a digital ruler in Adobe Photoshop 6.0 (Adobe systems, CA, USA). The frequency of pollen germination was counted when pollen tubes just protruded from the germination aperture. More than 250 pollen grains and 45 pollen tubes were measured in each treatment. Experiments were repeated three times.

### Cytosolic-free Ca^2+^ detection in pollen using confocal laser-scanning microscopy

Pollens were evenly placed in petri dishes containing basal medium alone, or with 10 mg/L ALA, 10 mg/L ALA + 10^−3^ M Ca^2+^, 10 mg/L ALA + 10^−3^ M Ca^2+^ + 10^−3^ M EGTA, and incubated at 25°C at 100% humidity, in the dark for 1 h.Pollen Ca^2+^ variations were determined with fluorescent dye Fluo-3 AM. One microliter 1 mM Fluo-3 AM (dissolved in DMSO, Sigma) was added to 50 μL pollen solution and then placed in the dark at 4°C for 2 h. The mixed liquor was centrifuged at 5000 rpm at 4°C for 10 min. The sediment was then washed three times through resuspension and centrifugation with 50 μL basal medium to remove the excess Fluo-3 AM. After standing for 1 h at 20°C, the pollen tube [Ca^2+^]_cyt_ was monitored using a laser scanning confocal microscope (Carl Zesis 780, LSCM), with the following settings: ex = 488 nm, em = 493–598 nm, power 2%, zoom 4, scanning 7, frame 1024 × 1024, and Time-course and Photoshop software. At least five biological replicates were performed and three images taken for each biological replicate.

### Assay of Ca^2+^-ATPase activity

Measurement of Ca^2+^-ATPase activity was performed using the Ca^2+^-ATPase assay kit (Jiancheng Bioengineering Institute). After being cultured for 3 h, pollen solution from each treatment was centrifuged at 5000 rpm for 10 min. The pollen sediment was collected and homogenized using a mortar filled with liquid nitrogen. The homogenate was added to 200 μL normal saline for the determination of total protein content and Ca^2+^-ATPase activity. The amount of total proteins was quantified using the method of Bradford (1976), using bovine serum albumin as the standard, and Ca^2+^-ATPase activity was assayed following the manufacturer's instructions. Enzyme activity was expressed as μmol Pi·mg^−1^Protein·h^−1^. Three biological replicates were performed.

### Statistical analysis

All data were taken from at least three independent experiments. Statistical analysis was performed using SPSS statistical computer package (version 16.0 SPSS Inc. Chicago. IL). Data was compared with the control or among treatments by analysis of variance (ANOVA) to discriminate significant differences at *P* < 0.05 or *P* < 0.01 followed by least significant difference tests (LSD).

## Results

### ALA reduced pear fruit set at low concentrations

**Figure 1:**
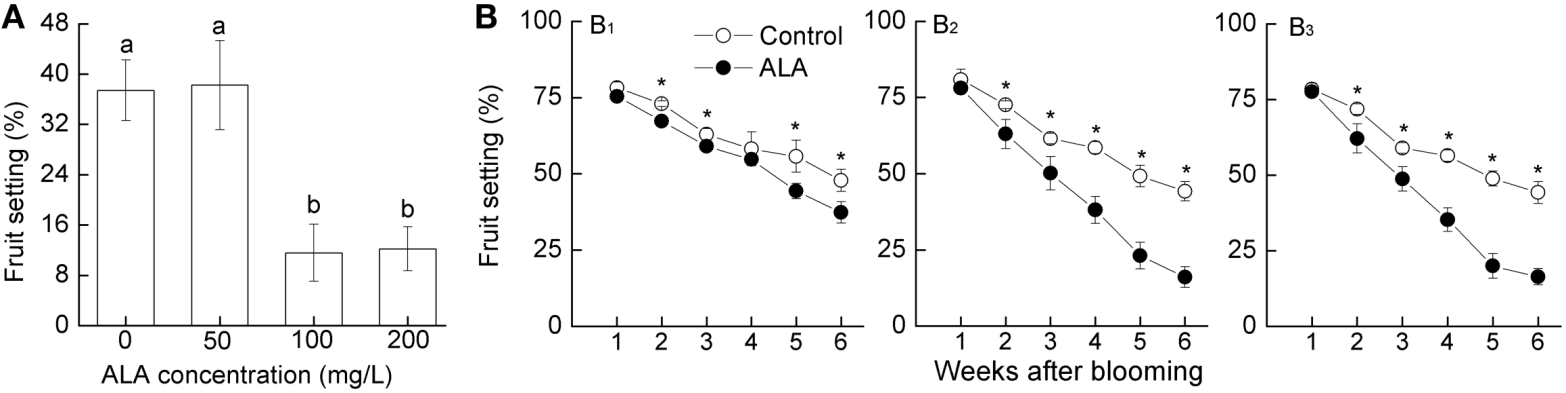
**ALA reduces pear (*Pyrus pyrifolia* Nakai) fruit set. (A)** Effects of ALA at different concentrations on pear fruit set. Exogenous ALA at 0, 50, 100, and 200 mg/L were sprayed evenly to the tagged branches, respectively, at 75% bloom. Fruit set for each treatment was recorded 6 weeks later to evaluate the appropriate concentration of exogenous ALA. **(B)** Timing effect of ALA on pear fruit set. At the 25 **(B_1_)**, 50 **(B_2_)**, or 75% **(B_3_)** bloom, exogenous ALA at 0 and 100 mg/L were sprayed evenly to the tagged branches, respectively. Fruit set for each treatment was recorded 6 weeks later to determine the optimum time for ALA application. Values are the means of 15 measurements ± SE from three independent experiments. Different small letters in **(A)** and ^*^ in **(B)** represent significant difference between treatments (*P* < 0.05).

Exogenously applying ALA at high concentrations (600–1200 mg/L) at the late of full bloom significantly thinned pear flowers and consequently reduced fruit set (Shen et al., 2011). In order to reduce the economic cost of ALA application, in the present study, we first investigated whether exogenously applying ALA at low concentrations at 75% bloom could thin fruits. Results showed that 100 and 200 mg/L ALA significantly reduced the later fruit set (to 11 and 12%, respectively), while 50 mg/L ALA had no obvious thinning effect (Figure 1A). Therefore, 100 mg/L was chosen as the appropriate concentration of ALA for pear fruit thinning.

To determine the optimal time for ALA application, we further investigated the effect of 100 mg/L ALA sprayed at different stages of blossom on fruit set. Results showed that application of 100 mg/L ALA at all tested stages significantly reduced the later fruit set (Figure 1B). However, compared with the application at 25% bloom (Figure 1B_1_), spraying ALA at 50 or 75% bloom thinned pear fruits more effectively, both of which reduced the fruit set to ~16% 6 weeks after blossom (Figures 1B_2_, B_3_). No visible detrimental effects on shoots and flower organs were found during the whole experiment. These results indicate that spaying 100 mg/L ALA at 50–75% bloom effectively thins pear flowers and fruits.

### ALA inhibited pollen tube growth in flowers of detached pear twigs

**Figure 2:**
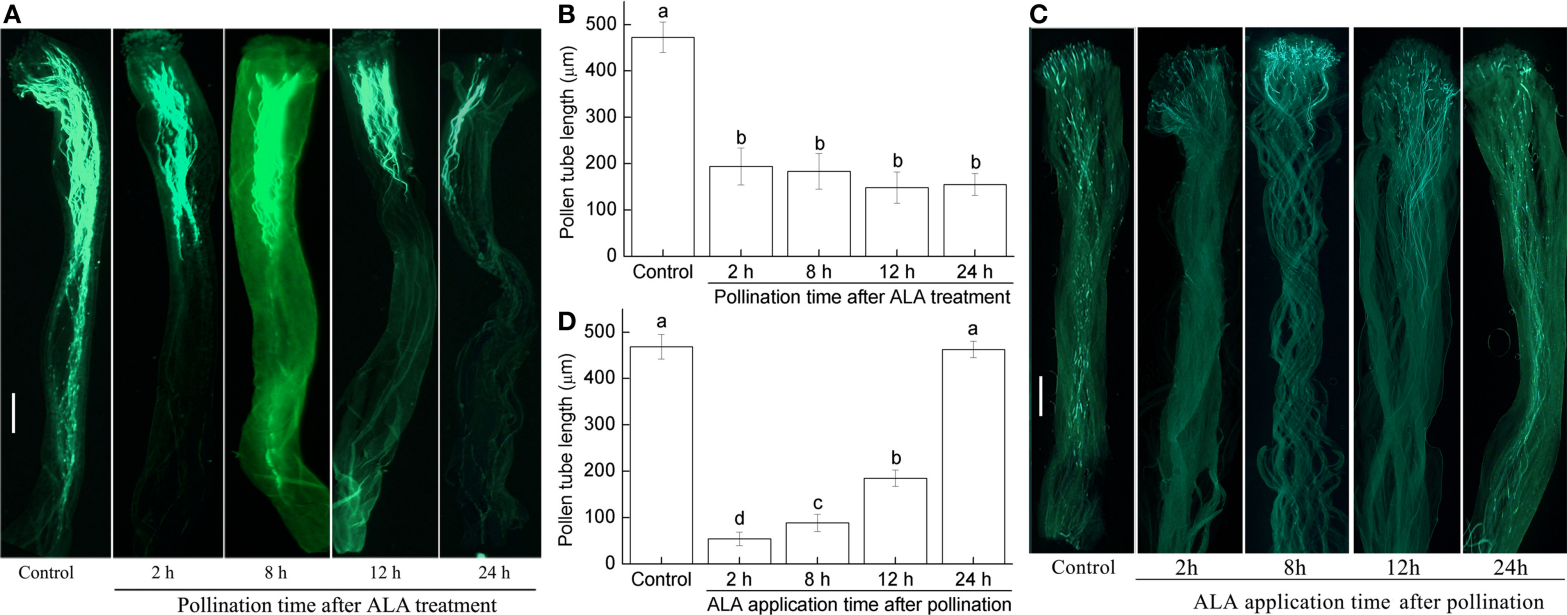
**ALA inhibits pollen tube growth of detached pear blossoms**. Flower twigs were cultured with 10% sucrose, in a growth chamber at 25°C under a PPFD of 50 μmol·m^−2^·s^−1^ in 12 h light/12 h dark cycles. **(A,B)** 100 mg/L ALA were sprayed to the flowers which were then hand pollinated 2, 8, 12, or 24 h later, respectively. Flowers that sprayed with distilled water and then hand pollinated 2, 8, 12, or 24 h later were set as controls. Because spraying water 2, 8, 12, and 24 h before pollination did not result in significant differences between pollen tube lengths, to make figure concise, only water application at 2 h before pollination was presented as Control. Fluorescence of the pollen tubes **(A)** at 48 h post-pollination was observed under a stereomicroscope (Olympus MVX10, Japan) and the pollen tube length **(B)** was determined. **(C,D)** flowers were hand pollinated and then sprayed 100 mg/L ALA 2, 8, 12, or 24 h later, respectively. Flowers that hand pollinated and then sprayed with distilled water 2, 8, 12, or 24 h later were set as controls. Because spraying water 2, 8, 12, and 24 h after pollination did not result in significant differences between pollen tube lengths, to make figure concise, only water application at 2 h after pollination was presented as Control. Fluorescence of the pollen tubes **(C)** at 48 h post-pollination was observed under a stereomicroscope (Olympus MVX10, Japan) and the pollen tube length **(D)** was determined. Scale bar: 50 μm. The white arrow points to the end of the pollen tubes. At least 15 styles from a population of three different plants were used for each treatment and experiments were repeated three times. Different small letters in **(B)** or **(D)** represent significant difference between treatments (*P* < 0.05).

To elucidate the mechanism underlying the thinning effect of ALA, 100 mg/L ALA was sprayed to the styles of “Cuiguan” flowers of detached twigs 2, 8, 12, and 24 h before (experiment I) or after (experiment II) hand pollination with “Akemizu” pollens. In experiment I, at 48 h post-pollination, most pollen tubes of the untreated control reached the ovary (Figure 2A), whereas nearly all ALA pretreated pollen tube growth only reached about one third of the length of the controls (Figure 2A). Specifically, spraying ALA 24, 12, 8, and 2 h before hand pollination significantly reduced the pollen tube length by 67, 69, 65, and 61%, respectively, compared with the controls (Figure 2B). These results indicated that if the flowers are pollinated within 24 h after ALA treatment, the pollen tube growth along the style will be significantly inhibited.

In experiment II, ALA spraying within 12 h after pollination also reduced the pollen tube length significantly, but ALA treatment 24 h after pollination showed no obvious inhibitory effect on pollen tube growth (Figure 2C). When applying ALA at 2, 8, and 12 h after pollination, the pollen tube length at 48 h post-pollination were only 11, 19, and 29% of that of the control, respectively (Figure 2D), suggesting that the earlier the ALA is applied, the shorter the tube length is. These results indicated that the pollen tube growth is blocked by ALA treatment within 12 h after pollination, but not affected after 24 h of pollination.

These results together indicate that exogenously applying 100 mg/L ALA between 24 h before pollination and 12 h after pollination significantly inhibits pollen tube growth, which supports the function of ALA-induced fruit thinning.

### ALA inhibited pollen germination and tube growth *in vitro*

We further examined the effect of exogenous ALA on pollen germination and tube growth *in vitro* by adding ALA to the culture medium. Generally, pollens are more sensitive to exogenous chemical reagents *in vitro*. Therefore, in the following *in vitro* experiments, the concentration of exogenous ALA was adjusted to 5–20 mg/L.

**Figure 3:**
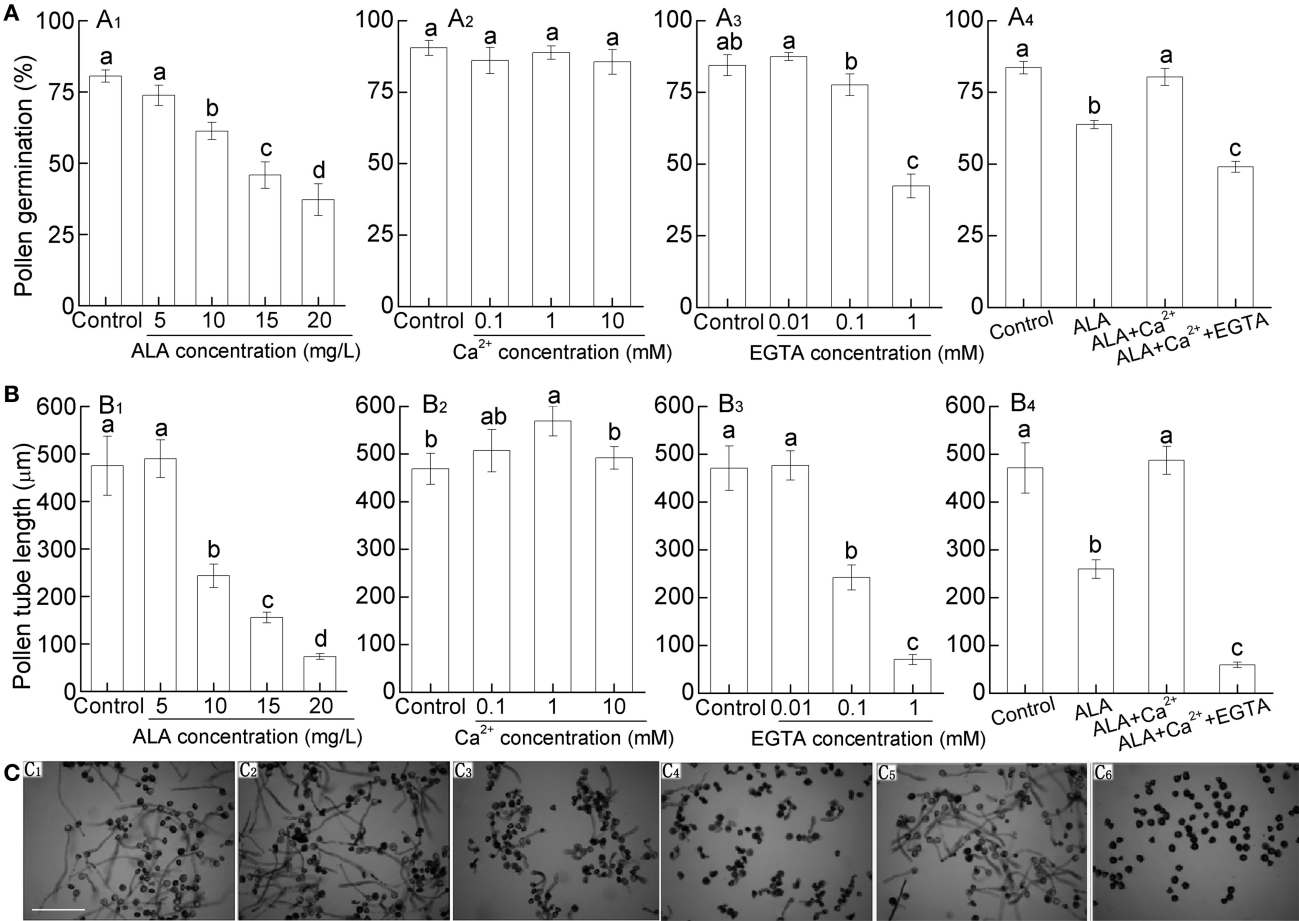
**ALA inhibits pollen germination and pollen tube length *in vitro* and this process might associate with calcium signaling**. Pollens were germinated in simplified culture, containing 10% sucrose and 0.01% H_3_BO_3_, or with treatment reagent, and incubated at 25°C at 100% humidity, in the dark for 3 h. **(A_1_, B_1_, C_1_–C_4_)** ALA inhibits pollen germination **(A_1_)** and pollen tube length **(B_1_)** *in vitro*. Images **(C_1_–C_4_)** represent pollen germination in simplified culture alone **(C_1_)**, or with 5 mg/L ALA **(C_2_)**, 10 mg/L ALA **(C_3_)**, and 20 mg/L ALA **(C_4_)**. **(A_2_,A_3_, B_2_,B_3_)** Effects of CaCl_2_ and EGTA at different concentrations on pollen germination **(A_2_,A_3_)** and pollen tube length **(B_2_,B_3_)**. **(A_4_,B_4_,C_5_,C_6_)** Reciprocal effect of 10 mg/L ALA, 10^−3^ M Ca^2+^, and 10^−3^ M EGTA on pollen germination **(A_4_)** and pollen tube length **(B_4_)**. **(C_5_,C_6_)** represent pollen germination in simplified culture with 10 mg/L ALA + 10^−3^ M Ca^2+^ **(C_5_)**, or 10 mg/L ALA + 10^−3^ M Ca^2+^ + 10^−3^ M EGTA **(C_6_)**. Images **(C)** were recorded under a light microscope (Nikon TE100, 400 ×), using a fitted camera (MShot Digital Imaging System), and pollen germination **(A)** and pollen tube length **(B)** were determined. Scale bar: 300 μm. More than 250 pollen grains and 45 pollen tubes were measured in each treatment and experiments were repeated three times. Different small letters in each figure represent significant difference between treatments (*P* < 0.05).

Application of 10–20 mg/L ALA significantly reduced pear pollen germination, while 5 mg/L ALA did not cause significant reduction (Figures 3A_1_,C_1_–C_4_). Similar to pollen germination rate, pollen tube growth was significantly inhibited under 10–20 mg/L ALA treatment (Figures 3B_1_,C_1_–C_4_). Since cytosolic free Ca^2+^ ([Ca^2+^]_cyt_) plays a pivotal role in controlling pollen germination and pollen tube elongation (Iwano et al., 2009), we next investigated whether ALA inhibited pollen germination and pollen tube elongation through regulating pollen [Ca^2+^]_cyt_. To determine the optimal concentration of Ca^2+^ and EGTA (a Ca-chelating agent), we observed the pollen germination rate and pollen tube growth under different concentrations of Ca^2+^ or EGTA. We found that 1 mM Ca^2+^ was the most effective in improving pollen tube growth (Figures 3A_2_,B_2_), and 1 mM EGTA inhibited pollen germination and tube length significantly (Figures 3A_3_,B_3_). Based on the results, 10 mg/L ALA, 1 mM Ca^2+^, and 1mM EGTA were chosen for the following experiments.

ALA alone significantly reduced pollen germination and pollen tube length (Figures 3A_4_,B_4_). However, when ALA was applied together with Ca^2+^, the inhibitory effect of ALA was disappeared (Figures 3A_4_,B_4_,C_5_). When EGTA was also added to the treatment solution, pollen germination, and pollen tube length were inhibited as ALA treatment alone (Figures 3A_4_,B_4_,C_6_). These results indicate that the inhibitory effect of ALA on pollen germination and pollen tube length is associated with the decrease of [Ca^2+^]_cyt_ in pollens.

### ALA reduced [Ca^2+^]_cyt_ concentration in pollens

**Figure 4:**
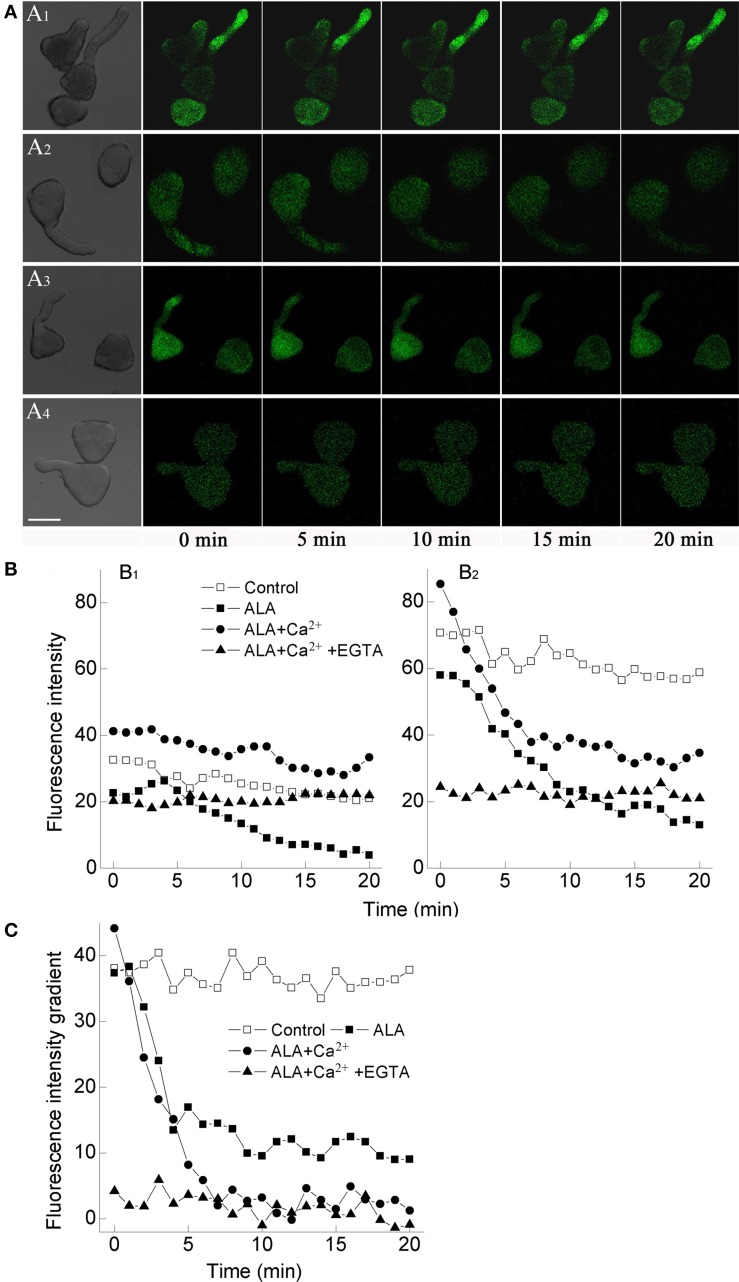
**ALA reduces cytosolic Ca^2+^ concentration and tip-focused Ca^2+^ gradient in pollens**. Pollens were evenly placed in petri dishes containing basal medium alone **(A_1_)**, or with 10 mg/L ALA **(A_2_)**, 10 mg/L ALA + 10^−3^ M Ca^2+^ **(A_3_)**, 10 mg/L ALA + 10^−3^ M Ca^2+^ + 10^−3^ M EGTA **(A_4_)**, and incubated at 25°C at 100% humidity, in the dark for 1 h. Then pollen solutions were loaded with 1 μM Fluo-3 AM (dissolved in DMSO) for 2 h in darkness at 4°C, then excess dye was removed. The fluorescence of the above-treated pollens **(A)** was observed using a laser scanning confocal microscope (Carl Zesis 780, LSCM) and Time-course and Photoshop software. For each treatment, the first picture is bright field image and the following are fluorescence images corresponding to the bright field image at 5 min intervals. Scale bar: 30 μm. **(B)** Time course changes of the relative fluorescence in the middle **(B_1_)** and the tip **(B_2_)** of pollen tubes. **(C)** Time course changes of the fluorescence intensity gradient between the tip and the middle pollen tube. Values are the means of 15 measurements ± SE from three independent experiments.

To confirm whether ALA functioned through reducing [Ca^2+^]_cyt_, we determined [Ca^2+^]_cyt_ in germinating pollens directly using a fluorescent dye, Fluo-3 AM. Compared with the control group, ALA significantly reduced [Ca^2+^]_cyt_ in pollens and pollen tubes as indicated by the decrease of fluorescence intensity (Figure 4A_2_). When ALA and Ca^2+^ were applied together, [Ca^2+^]_cyt_ was strongly increased as compared to ALA treatment alone (Figure 4A_3_). When EGTA was applied together with ALA and Ca^2+^, [Ca^2+^]_cyt_ decreased again (Figure 4A_4_).

Since the tip-specific [Ca^2+^]_cyt_ gradient plays a pivotal role in controlling pollen tube elongation (Wu et al., 2010), we further compared the effects of ALA on [Ca^2+^]_cyt_ in the middle and the tip of pollen tube. We found that fluorescence intensity in the tip (Figure 4B_2_) was more significantly reduced than that in the middle (Figure 4B_1_) upon ALA treatment, which was more visually reflected by the fluorescence intensity gradient between the tip and the middle pollen tube (Figure 4C). Compared to the steep gradient in the control pollen tube, the fluorescence intensity gradient became gentler under ALA treatment as time went on (Figure 4C). Similar trend of the intensity gradient was also observed under the combined treatment of ALA and Ca^2+^, which further proved the reducing effect of ALA on “tip-focused” [Ca^2+^]_cyt_ gradient.

### ALA promoted the Ca^2+^-ATPase activity in pollens

**Figure 5:**
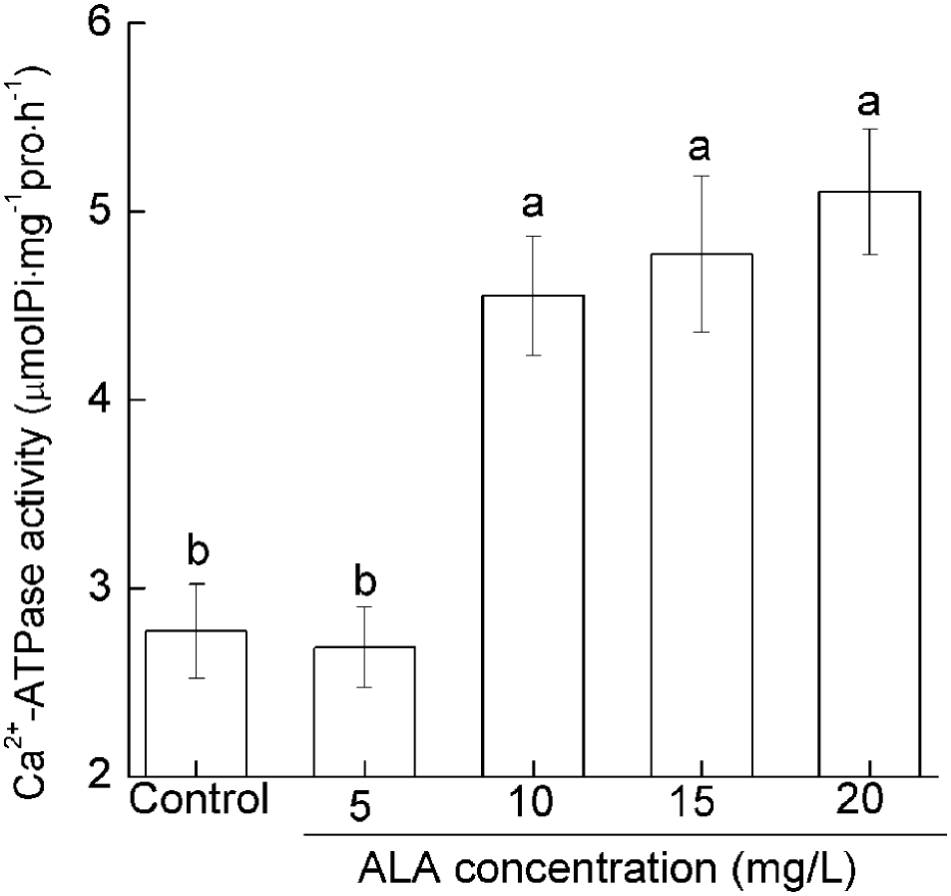
**ALA promotes the pollen Ca^2+^-ATPase activity**. Pollens were germinated in simplified culture, containing 10% sucrose and 0.01% H_3_BO_3_, or with 5–20 mg/L ALA, and incubated at 25°C at 100% humidity, in the dark for 3 h. Then Ca^2+^-ATPase activity was assayed using the Ca^2+^-ATPase assay kit. Values are the means of 9 measurements ± SE from three independent experiments. Different small letters represent significant difference between treatments (*P* < 0.05).

To determine how ALA regulates pollen [Ca^2+^]_cyt_, we investigated the effect of ALA on pollen Ca^2+^-ATPase activity which plays important roles in the efflux of Ca^2+^ (Guan et al., 2013). ALA at 5 mg/L showed no significant influence on pollen Ca^2+^-ATPase activity. However, 10–20 mg/L ALA promoted pollen Ca^2+^-ATPase activity drastically (Figure 5). Under 10, 15, and 20 mg/L ALA treatment, Ca^2+^-ATPase activity increased by 84.20, 72.24, and 64.20% of the control, respectively (Figure 5). These results indicated that ALA-induced reduction of [Ca^2+^]_cyt_ is probably achieved through the activation of Ca^2+^-ATPase.

### Pollen germination and tube elongation were negatively correlated with Ca^2+^-ATPase activity

**Figure 6:**
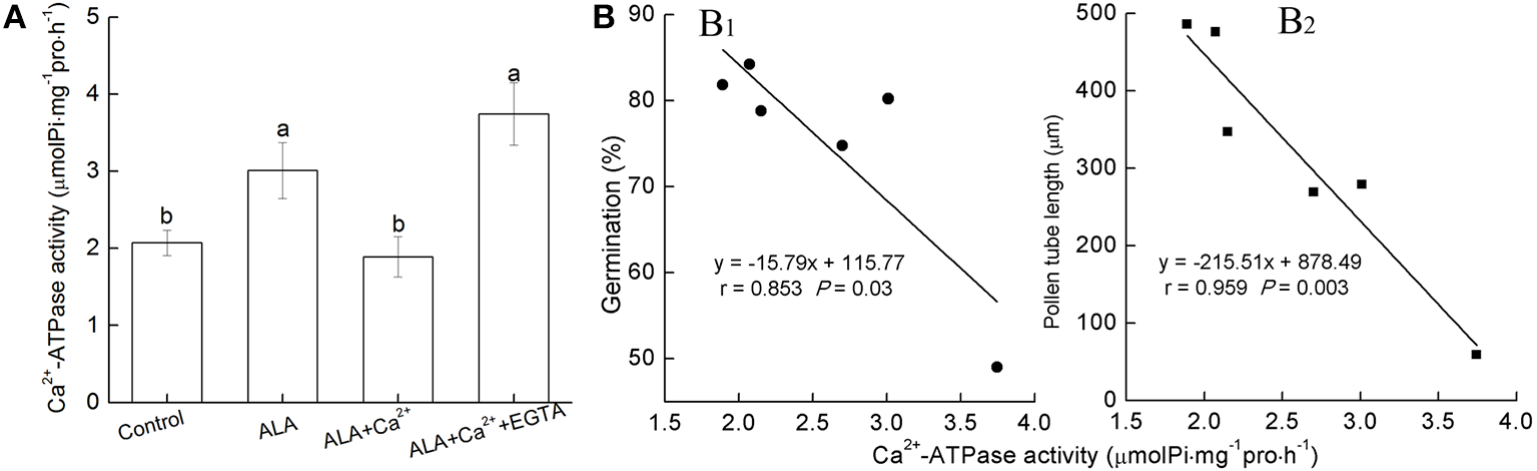
**Correlation between Ca^2+^-ATPase activity and pollen germination rate or pollen tube length**. **(A)** Reciprocal effect of ALA on pollen Ca^2+^-ATPase activity. Pollens were evenly placed in petri dishes containing basal medium alone, or with 10 mg/L ALA, 10 mg/L ALA + 10^−3^ M Ca^2+^, 10 mg/L ALA + 10^−3^ M Ca^2+^ + 10^−3^ M EGTA, and incubated at 25°C at 100% humidity, in the dark for 3 h. Then Ca^2+^-ATPase activity was assayed using the Ca^2+^-ATPase assay kit. **(B)** Negative correlation between Ca^2+^-ATPase activity and pollen germination rate **(B_1_)** or pollen tube length **(B_2_)**. Pollens were evenly placed in petri dishes containing basal medium alone, or with 10 mg/L ALA, 10 mg/L ALA + 10^−5^ M Ca^2+^, 10 mg/L ALA + 10^−4^ M Ca^2+^, 10 mg/L ALA + 10^−3^ M Ca^2+^, 10 mg/L ALA + 10^−3^ M Ca^2+^ + 10^−3^ M EGTA, and incubated at 25°C at 100% humidity, in the dark for 3 h. Then pollen germination rate, pollen tube length, and Ca^2+^-ATPase activity were determined and correlation analysis between them were carried out. Values are the means of 9 measurements ± SE from three independent experiments.

We next investigated Ca^2+^-ATPase activity in response to ALA, Ca^2+^, and EGTA treatment (Figure 6A). When exposed to ALA alone, pollen Ca^2+^-ATPase activity significantly increased. When ALA was applied together with Ca^2+^, pollen Ca^2+^-ATPase activity remained at the level as control treatment. In the presence of EGTA, Ca^2+^-ATPase activity increased to the level as ALA treatment alone. Therefore, it seemed that Ca^2+^-ATPase activity was correlated with ALA effects. To further reveal roles of Ca^2+^-ATPase in ALA-inhibited pollen germination and tube growth, correlation analyses between pollen Ca^2+^-ATPase activity and pollen germination or pollen tube length were conducted (Figure 6B). The significant negative correlation between pollen Ca^2+^-ATPase activity and pollen germination or pollen tube length further supported the role of Ca^2+^-ATPase in ALA-inhibited pollen germination and tube growth.

## Discussion

Many fruit trees, such as pear, apple, and peach trees, usually overset, even after poor pollination conditions. Due to an unsatisfactory leaf/fruit ratio, an excessive crop often adversely affect fruit size, color, sugars, and other quality components, and fruit storage potential (Bangerth, 2000). Therefore, flower or fruit thinning has become necessary in modern fruit production. Since manual thinning is neither practical nor economical when thinning large numbers of trees, chemical thinning including thinning with bioregulators has become the inevitable alternative (Bangerth, 2000; Gonkiewicz et al., 2011). ALA is a natural plant growth regulator, which has been proved to be remarkably effective in improving plant photosynthesis, abiotic stress, and fruit quality (Akram and Ashraf, 2013). Shen et al. (2011) firstly showed that exogenous ALA at 600–1200 mg/L thinned “Hosui” pear fruits and improved fruit quality. This report suggested that ALA is a potential chemical thinner which might be used in pear production. It is exciting that ALA can be used as a biological thinner, as ALA is readily biodegradable and nontoxic to mammals, and has no adverse effects on the environment (Sasikala et al., 1994). However, in that study, the application concentrations of ALA were too high to implement due to the high cost. Moreover, high concentrations of ALA thinned flowers mainly through activating photodynamic damage on flower organs including the stigma (Shen et al., 2011), similar to its role as a photodynamic herbicide (Sasikala et al., 1994). Here, we showed that ALA at 100–200 mg/L could thin “Cuiguan” pear fruits effectively (to 11–16%) when sprayed at 50–75% bloom, and no visible detrimental effects on flowers or leaves were found. The 11–16% fruit set is in the range of ideal pear fruit set percentage (Ohkawa et al., 2006). Furthermore, a similar thinning effect of ALA at 100–200 mg/L was found in other pear cultivars such as “Akemizu” and “Suisho” (Supplementary Figure S1), implying that the thinning effect of ALA is universal across pear varieties.

A feasible chemical thinner should not adversely affect fruit weight and quality while reducing fruit set. Although we did not measure fruit quality in this study, we have previously demonstrated that exogenous ALA solutions from low concentration (75 mg/L) to high concentration (1200 mg/L) significantly improved photosynthetic capacity and fruit quality of various fruit trees, such as “Hosui” pear (*P. pyrifolia*) (Shen et al., 2011), “Fuji” apple (*Malus dometica*; Xie et al., 2013a), and “Summer Black” grape (*Vitis vinifera*; Xie et al., 2013b). Consistently, other researchers also reported the positive effects of ALA at 80–250 mg/L on fruit weight, size, and quality of strawberry (Iwai et al., 2005), grape (*V. vinifera*; Watanabe et al., 2006), date palm (*Phoenix dactylitera*; Al-Khateeb et al., 2006; Al-Qurashi and Awad, 2011), and lichi (*Lichi chinensis*; Feng et al., 2015). Therefore, the improvement of fruit weight and quality by ALA may be a common effect across fruit tree species, and ALA over a very wide range of concentrations exhibits this effect. These results indicate that ALA at low concentrations would improve fruit weight and quality while thinning fruits. ALA is a natural amino acid present in all living cells, and it is biodegradable and harmless for crops, humans, and animals (Sasikala et al., 1994). Therefore, fruit thinning with ALA at low concentrations is more likely to meet modern environmental and food quality guidelines, suggesting its great application potential in modern fruit production.

Fruit thinning at the appropriate time allows the remaining fruit to develop to its maximum size. Thinning at bloom time often results in larger fruits at harvest, whereas thinning fruits too late reduces the chances that fruit size will increase (Miller and Tworkoski, 2010). Here, ALA application at 50–75% bloom effectively thinned fruits. Many chemical thinners thin fruits by preventing fertilization (Ohkawa et al., 2006). Pollen germination and tube growth are critical for the delivery of sperm cells to the ovule, leading to successful fertilization (Krichevsky et al., 2007). Ca-formate at high concentrations can thin pear flowers, but causes injuries to sprouting shoots and petals, while low concentrations (1–3%) can also thin flowers but mainly through inhibiting pollen germination and tube growth (Hiratsuka et al., 2003). Similarly, ALA at high concentration can thin pear flowers mainly through causing photodynamic damage to flower organs (Shen et al., 2011). However, in the present study, applying low concentrations of ALA to the open flowers before pollination or within 12 h after pollination significantly inhibits the pollen tube growth, with no visible injury on the flower organs. These results indicated that ALA at low concentrations thin fruits by preventing fertilization through inhibiting pollen tube elongation, which was further confirmed by *in vitro* pollen germination experiment (Figure 3). Moreover, inhibitory effect of ALA on pollen tube growth was observed only when ALA was sprayed to the open flowers before pollination or within 12 h after pollination. This characteristic makes the application time of ALA more explicit for growers in the actual production.

Pollen tube growth is tightly regulated both spatially and temporally (Franklin-Tong, 1999). [Ca^2+^]_cyt_ is an important secondary messenger regulating pollen tube growth (Lazzaro et al., 2005; Guan et al., 2013; Steinhorst and Kudla, 2013). In our study, the inhibitory effects of ALA on pollen germination and pollen tube length were totally prevented by the addition of Ca^2+^, suggesting that ALA inhibits pollen tube growth by decreasing [Ca^2+^]_cyt_ in pollen tubes. EGTA is a Ca-chelating agent which is often used as a negative regulator to analyze physiological roles of Ca^2+^. When EGTA and Ca^2+^ were applied together to the culture medium with ALA, the inhibitory effect of Ca^2+^ on ALA-induced reduction of pollen germination and tube growth was reversed, further indicating the involvement of Ca^2+^ signal in this process. It has been well established that a positive correlation exists between changes in [Ca^2+^]_cyt_ and pollen tube growth rate (Franklin-Tong, 1999). Here, ALA significantly reduced [Ca^2+^]_cyt_ in pollen tubes, providing direct evidence that inhibitory effect of ALA on pollen tube growth results from the decreasing [Ca^2+^]_cyt_ in pollen tubes. Not only high concentration of [Ca^2+^]_cyt_ but also the tip-focused [Ca^2+^]_cyt_ gradient are required for the growth of pollen tubes (Pierson et al., 1994; Wu et al., 2010). We showed that ALA also significantly dissipated the tip-focused [Ca^2+^]_cyt_ gradient in pollen tubes, further suggesting the negative effect of ALA on Ca^2+^ signal in pollen tubes.

The mechanisms behind the regulation of tip-focused Ca^2+^ gradients are complex, involving many components and potential pathways that increase or decrease the cytosolic Ca^2+^ concentration. Ca^2+^-ATPase plays an important role in the negative regulation of tip Ca^2+^ gradient by not only involving the efflux of Ca^2+^ on the plasma membrane, but also sequestering Ca^2+^ within endomembrane system (Guan et al., 2013). In the present study, ALA at 10–20 mg/L, which significantly inhibited pollen tube growth, substantially promoted Ca^2+^-ATPase activity. Therefore, ALA-induced decrease of [Ca^2+^]_cyt_ and tip-focused [Ca^2+^]_cyt_ gradient probably results from the activation of Ca^2+^-ATPase. The significant correlations between Ca^2+^-ATPase activity and pollen germination rate or pollen tube length further suggest the critical role of calcium pump in ALA's negative effect on pollen germination. Taken together, we conclude that thinning effect of ALA results from Ca^2+^-ATPase-mediated Ca^2+^ efflux from pollen tubes. In fact, the thinning effect of ALA lasted for several weeks after blossom, indicating that ALA may also thin fruits through other pathways besides prevention of fertilization by inhibiting pollen tube growth.

ALA shows multi-physiological functions and has been recognized as a new plant growth regulator. However, the functional mechanisms behind its various physiological roles are largely unknown. Our previous study showed that Ca^2+^ signal plays an important role in ALA-induced stomatal opening (Chen et al., 2014). In the present study, we also demonstrated that Ca^2+^ signal mediates the inhibitory effect of ALA on pollen germination and tube growth. Therefore, Ca^2+^ may be a central signaling component in many physiological processes mediated by ALA.

## Conclusions

In conclusion, spraying 100–200 mg/L ALA to pear flowers at 50–75% bloom significantly thinned pear fruits without detrimental effects. We showed that ALA at low concentrations inhibited pollen germination and tube growth, leading to fertilization failure. The Ca^2+^ efflux from pollen tubes mediated by Ca^2+^-ATPase played crucial roles in the negative effects of ALA on pollen germination. Although it cannot be excluded that ALA at low concentrations thin fruits through other pathways, our results suggest ALA as an ideal biochemical thinner for modern fruit production, presenting a new role for ALA. The results, especially the regulatory role of ALA in Ca^2+^ signal, also provide directions for further research about the functional mechanisms behind various physiological roles of ALA.

## Author contributions

YA, LW conceived and designed research. JL, CD carried out all the experiments. YA, JL, LL analyzed the data. YS, RC contributed to reagents, materials, and analysis tools. YA, LW wrote the manuscript. All authors read and approved the manuscript.

## Funding

This research was supported by the National Science Foundation of China (31401820), the Fundamental Research Funds for the Central Universities (KJQN201538), the Natural Science Foundation of Jiangsu Province, China (BK20140702), and Agricultural Independent Innovation Fund of Jiangsu Province, China [CX(11)4004]. The funders had no role in study design, data collection and analysis, decision to publish, or preparation of the manuscript.

### Conflict of interest statement

The authors declare that the research was conducted in the absence of any commercial or financial relationships that could be construed as a potential conflict of interest.

## References

[B1] Akram N. A. Ashraf M. (2013). Regulation in plant stress tolerance by a potential plant growth regulator, 5-aminolevulinic acid. J. Plant Growth Regul. 32, 663–679. 10.1007/s00344-013-9325-9

[B2] Al-Khateeb A. A. Al-Khateeb S. A. Lkawara R. Al-abdoulhady I. A. (2006). Promotive effects of 5-aminolevulinic acid (5-ALA) on fruit yield and quality of date palm cv., Khalas. J. Biol. Sci. 6, 1118–1121. 10.3923/jbs.2006.1118.1121

[B3] Al-Qurashi A. D. Awad M. A. (2011). 5-Aminolevulinc acid increases tree yield and improves fruit quality of ‘Rabia’ and ‘Sukkariat-Yanbo’ date palm cultivars under hot arid climate. Sci. Hort. Amsterdam 129, 441–448. 10.1016/j.scienta.2011.04.014

[B4] Bangerth F. (2000). Abscission and thinning of young fruit and thier regulation by plant hormones and bioregulators. Plant Growth Regul. 31, 43–59. 10.1023/A:1006398513703

[B5] Bound S. A. (2006). Comparison of two 6-benzyladenine formulations and carbaryl for post-bloom thinning of apples. Sci. Hort. Amsterdam 111, 30–37. 10.1016/j.scienta.2006.07.028

[B6] Bradford M. M. (1976). A rapid and sensitive method for the quantitation of microgram quantities of protein utilizing the principle of protein-dye bingding. Anal. Biochem. 72, 248–254. 10.1016/0003-2697(76)90527-3 942051

[B7] Burge G. K. Spence C. B. Dobson B. G. (1991). The response of Hosui Japanese pear to time of hand thinning and chemical thinning agents. Sci. Hort. Amsterdam 45, 245–250. 10.1016/0304-4238(91)90069-B

[B8] Chen L. H. Liu L. B. An Y. Y. Zhang Z. P. Wang L. J. (2014). Preliminary studies on the possible mechanism underlying 5-aminolevulinic acid-induced stomatal opening in apple leaves. Acta Hort. Sin. 41, 1965–1974 (in Chinese with abstract in English).

[B9] Costa G. Vizzotto G. Malossini C. Ramina A. (1995). Biological activity of a new chemical agent for peach flower thinning. Acta Hort. 394, 123–128. 10.17660/ActaHortic.1995.394.11

[B10] Feng S. Li M. F. Wu F. Li W. L. Li S. P. (2015). 5-Aminolevulinic acid affects fruit coloration, growth, and nutrition quality of *Litchi chinensis* Sonn. cv. Feizixiao in Hainan, tropical China. Sci. Hort. Amsterdam 193, 188–194. 10.1016/j.scienta.2015.07.010

[B11] Franklin-Tong V. E. (1999). Signaling and the modulation of pollen tube growth. Plant Cell 11, 727–738. 10.1105/tpc.11.4.727 10213789 PMC144203

[B12] Gonkiewicz A. Blaszczyk J. Basak A. (2011). Chemical pear fruit thinning. J. Fruit Ornam. Plant Res. 19, 73–78.

[B13] Guan Y. F. Guo J. Z. Li H. Yang Z. B. (2013). Signaling in pollen tube growth: crosstalk, feedback, and missing links. Mol. Plant 6, 1053–1064. 10.1093/mp/sst070 23873928 PMC3842152

[B14] Hiratsuka S. Niwa K. Kawai Y. Maejima T. Kawamura K. (2003). Flower thinning by calcium formate in Japanese pears: concentration effects and its uptake into styles. J. Jpn. Soc. Hortic. Sci. 72, 224–229. 10.2503/jjshs.72.224

[B15] Iwai K. Saito A. van Leeuwen J. Tanaka T. Takeuchi Y. (2005). A new functional fertilizer containing 5-aminolevulinic acid promoted hydroponically-grown vegetables in the Netherlands. Acta Hort. 697, 351–355. 10.17660/ActaHortic.2005.697.44

[B16] Iwano M. Entani T. Shiba H. Kakita M. Nagai T. Mizuno H.. (2009). Fine-tuning of the cytoplasmic Ca^2+^ concentration is essential for pollen tube growth. Plant Physiol. 150, 1322–1334. 10.1104/pp.109.139329 19474213 PMC2705041

[B17] Janoudi A. Flore J. A. (2005). Application of ammonium thiosulfate for blossom thinning in apples. Sci. Hort. Amsterdam 104, 161–168. 10.1016/j.scienta.2004.08.016

[B18] Jones K. M. Koen T. B. Oakford M. J. Bound S. (1989). Thinning red Fuji apples with ethephon or NAA. J. Hort. Sci. 64, 527–532. 26826356

[B19] Krichevsky A. Kozlovsky S. V. Tian G. W. Chen M. H. Zaltsman A. Citovsky V. (2007). How pollen tubes grow. Dev. Biol. 303, 405–420. 10.1016/j.ydbio.2006.12.003 17214979

[B20] Lazzaro M. D. Cardenas L. Bhatt A. P. Justus C. D. Phillips M. S. Holdaway-Clarke T. L.. (2005). Calcium gradients in conifer pollen tubes; dynamic properties differ from those seen in angiosperms. J. Exp. Bot. 56, 2619–2628. 10.1093/jxb/eri256 16118258

[B21] Miller S. S. Tworkoski T. (2010). Blossom thinning in apple and peach with an essential oil. HortScience 45, 1218–1225.

[B22] Ohkawa K. Ohara H. Kurita Y. Fuk-Uda T. Khan Z. U. Matsui H. (2006). Thinning effect of jasmonic acid derivative, n-propyl dihydrojasmonate on Japanese pear ‘Hosui’. J. Jpn. Soc. Hort. Sci. 75, 129–134. 10.2503/jjshs.75.129

[B23] Pierson E. S. Miller D. D. Callaham D. A. Shipley A. M. Rivers B. A. Cresti M.. (1994). Pollen-tube growth is coupled to the extracellular calcium-ion flux and the intracellular calcium gradient - effect of BAPTA-type buffers and hypertonic media. Plant Cell 6, 1815–1828. 10.1105/tpc.6.12.1815 7866026 PMC160564

[B24] Sasikala C. Ramana C. V. Rao P. R. (1994). 5-Aminolevulinic acid-a potential herbicide insecticide from microorganisms. Biotechnol. Progr. 10, 451–459. 10.1021/bp00029a001

[B25] Schiøtt M. Romanowsky S. M. Baekgaard L. Jakobsen M. K. Palmgren M. G. Harper J. F. (2004). A plant plasma membrane Ca^2+^ pump is required for normal pollen tube growth and fertilization. Proc. Nat. Acad. Sci. U.S.A. 101, 9502–9507. 10.1073/pnas.0401542101 15197266 PMC439006

[B26] Shen M. Duan C. H. Zhang Z. P. Cheng Y. Wang L. J. Li B. J. (2011). Effects of exogenous ALA on thinning and fruit quality in ‘Hosui’ pear (*Pyrus pyrifolia*). Acta Hort. Sin. 38, 1515–1522 (in Chinses with abstract in English).

[B27] Steinhorst L. Kudla J. (2013). Calcium - a central regulator of pollen germination and tube growth. Biochim. Biophys. Acta 1833, 1573–1581. 10.1016/j.bbamcr.2012.10.009 23072967

[B28] Stopar M. (2002). Thinning of ‘Gala’ and ‘Golden Delicious’ apples with BA, NAA and their combinations. J.Cent. Eur. Agr. 3, 1–6.

[B29] Watanabe K. Nishihara E. Watanabe S. Tanaka T. Takahashi K. Takeuchi Y. (2006). Enhancement of growth and fruit maturity in 2-year-old grapevines cv. Delaware by 5-aminolevulinic acid. Plant Growth Regul. 49, 35–42. 10.1007/s10725-006-0024-4

[B30] Wu J. Y. Shang Z. L. Wu J. Jiang X. T. Moschou P. N. Sun W. D.. (2010). Spermidine oxidase-derived H_2_O_2_ regulates pollen plasma membrane hyperpolarization-activated Ca^2+^-permeable channels and pollen tube growth. Plant J. 63, 1042–1053. 10.1111/j.1365-313XX.2010.04301.x 20626657

[B31] Xie L. Cheng X. H. Feng X. X. Yang T. Zhang Z. P. Wang L. J. (2013b). Effects of an amino acid fertilizer on the leaf photosynthesis and fruit quality of ‘Summer Black’ grape. *J. Nanjing Agric*. Univ. 36, 31–37 (in Chinses with abstract in English).

[B32] Xie L. Wang Z. H. Cheng X. H. Gao J. J. Zhang Z. P. Wang L. J. (2013a). 5-Aminolevulinic acid promotes anthocyanin accumulation in Fuji apples. Plant Growth Regul. 69, 295–303. 10.1007/s10725-012-9772-5

