# Supplementary material for: 5-Aminolevulinic Acid Thins Pear Fruits by Inhibiting Pollen Tube Growth via Ca2+-ATPase-Mediated Ca2+ Efflux
Source: Front Plant Sci. 2016 Feb 9;7:121. doi: 10.3389/fpls.2016.00121 (PMC4746310; doi:10.3389/fpls.2016.00121)
Supplement: Supplementary file 1 [file DataSheet1.DOC]

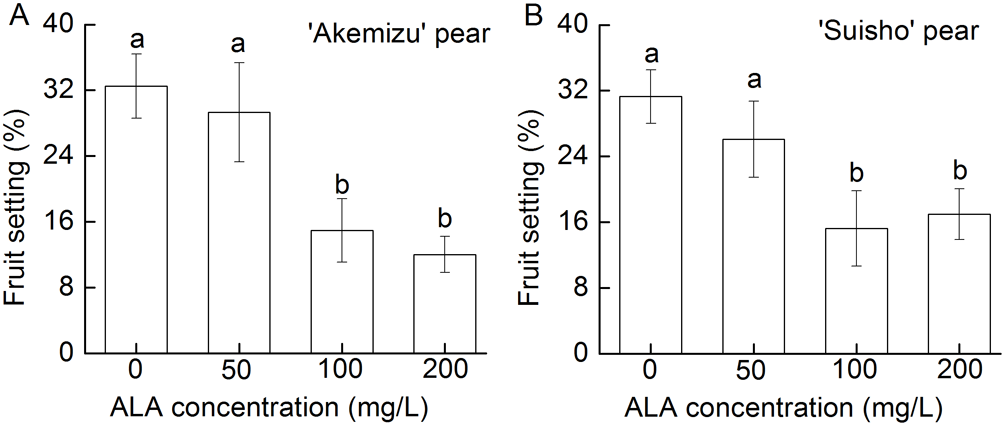


**Supplementary Figure S1│ ALA reduces fruit set of ‘Akemizu’ (A) and ‘Suisho’ (B) pears (*Pyrus pyrifolia* Nakai).** Exogenous ALA at 0, 50, 100 and 200 mg/L were sprayed evenly to the tagged branches, respectively, at 75% bloom. Fruit set for each treatment was recorded six weeks later. Values are the means of 15 measurements ± SE from three independent experiments. Different small letters represent significant difference between treatments (*P*＜0.05).
